# Experimental bluetongue virus superinfection in calves previously immunized with bluetongue virus serotype 8

**DOI:** 10.1186/s13567-016-0357-6

**Published:** 2016-07-28

**Authors:** Ludovic Martinelle, Fabiana Dal Pozzo, Pierre Sarradin, Willem Van Campe, Ilse De Leeuw, Kris De Clercq, Christine Thys, Etienne Thiry, Claude Saegerman

**Affiliations:** 1 Research Unit in Epidemiology and Risk Analysis Applied to the Veterinary Sciences (UREAR-ULg), Department of Infectious and Parasitic Diseases, Center for Fundamental and Applied Research for Animal and Health (FARAH), Faculty of Veterinary Medicine, University of Liege, Quartier Vallée 2, Avenue de Cureghem 7A, B42, 4000 Liège, Belgium; 2 INRA, UE 1277, Experimental Infectiology Platform, INRA-Research Centre of Tours, Nouzilly, France; 3 Animal Experimental Centre, CODA-CERVA, Kerklaan 68, 1830 Machelen, Belgium; 4 Unit Vesicular and Exotic Diseases, CODA-CERVA, Groeselenberg 99, Uccle, 1180 Brussels, Belgium; 5 Veterinary Virology and Animal Viral Diseases, Department of Infectious and Parasitic Diseases, Faculty of Veterinary Medicine, Center for Fundamental and Applied Research for Animal and Health (FARAH), University of Liege, Quartier Vallée 2, Avenue de Cureghem 10, B43bis, 4000 Liège, Belgium

## Abstract

The effect of a superinfection with bluetongue virus serotype 1 (BTV1) was evaluated on two groups of four calves. One group received a commercial inactivated BTV serotype 8 (BTV8) vaccine. This group and the non-vaccinated group of calves were challenged twice (4 months apart) with the European BTV8 strain isolated during the 2006–2007 epidemics. Calves were then infected with a BTV1 inoculum which was found to be unexpectedly contaminated by BTV serotype 15 (BTV15). BTV1 and BTV15 single infections were performed on two other groups of three BTV naïve calves. A severe clinical picture was obtained after superinfection with BTV1/BTV15 in both vaccinated and non-vaccinated animals and after challenge with BTV8 in non-vaccinated animals. BTV1 and BTV15 single infection caused only very slight clinical signs. After superinfection and at the viraemic peak, there were an average of above 1000 times more BTV15 genomic copies than BTV1 ones. BTV1 RNA could be detected only in the spleen of one calf whereas BTV15 RNA was found in 15 organs of seven different animals. BTV8 immunization whether it was acquired through vaccination and challenges or challenges alone did not change BTV1 or BTV15 RNA detection in superinfected animals. However in these animals a partial cross neutralization between BTV8 and BTV1 might be involved in the lower BTV1 replication versus BTV15. Infection with different serotypes can occur also in the field. Interference between virus strains, genetic reassortment and cross-protection were considered as mechanisms to explain the clinical outcomes and the other virological and immunological findings in the course of BTV1/BTV15 superinfection.

**Electronic supplementary material:**

The online version of this article (doi:10.1186/s13567-016-0357-6) contains supplementary material, which is available to authorized users.

## Introduction

Bluetongue (BT) is a non-contagious disease affecting ruminants and is caused by the bluetongue virus (BTV), the type species of the genus *Orbivirus*. BT is a World Organization for Animal Health reportable disease and is of considerable socioeconomic concern and of major importance in the international trade of animals and animal products [1]. Economic losses associated with BTV infection are caused directly through reductions in animal productivity and death, implementation of control measures, and more importantly, indirectly through trade losses due to animal movement restrictions [2].

Within each different *Orbivirus* species, several virus serotypes are identified, based on the specificity of reactions with the neutralizing antibodies generated by their mammalian host [3]. These reactions are dependent to a large extent to VP2 and also VP5, which are the most variable proteins in BTV; VP2 especially contains the most epitopes that drive neutralizing antibodies production and therefore is the main determinant of the serotype [4]. To date, 27 serotypes have been identified, including BTV25 identified in Switzerland in 2007 [5], BTV26 from Kuwait in 2010 [4] and BTV27 detected in goats in Corsica (France) in 2014 [6]. In addition, two putative new serotypes, respectively BTV28 and BTV29 were recently detected [7]. Indeed serological cross-reactions between different serotypes are described [8] and evidences of possible heterologous cross-protection do exist [9, 10], but their influence on the epidemiology of the disease is not sufficiently understood.

From 2006 to 2015, seven BTV serotypes were detected in Western and Central Europe, namely BTV1, 6, 8, 11, 14, 25, and 27. Most of the economic losses have to be attributed to BTV8, and to a lesser extent, BTV1, with respectively over 27 000 and 6000 holdings affected only in 2008. BTV8 alone was responsible for the death of more than 20 000 sheep in Belgium, which represents 5–10% of the national flock [11]. The 2008 BTV8 epizootic in Northern Europe is believed to have caused greater economic damage than any previous single serotype BT outbreak [12]. By contrast re-emergence of BTV8 in France in 2015 was only of limited impact [13].

On the other hand, BTV1 that circulated contemporarily in Southwest Europe, was described as a virus leading to subclinical or mild disease in cattle [14]. As a consequence of this epidemiologic context, domestic ruminants in the field could be sequentially infected by these two serotypes, as it was reported in France and Spain [15].

In this paper the results of a 9 month-long experiment are shown. Calves were originally divided in two groups, with one group being vaccinated against BTV8, and were subsequently both challenged with a homologous BTV8 European strain. In order to mimic the occurrence of repetitive infections according to studies reporting several peaks of vector activity during the course of the year [16], the same calves were infected a second time with the same BTV8 strain and later with BTV serotype 1 (BTV1) (superinfection). The aim of this study was to analyse the outcome of these successive challenges, taking into account the influence of vaccine immunity as well as natural post-infection immunity. The BTV1 inoculum appeared to be contaminated with BTV15 [17]. In order to evaluate any in vivo cross-protection, the consequences of a BTV1 and BTV15 single infection in BTV naïve calves were also considered.

## Materials and methods

### BTV8 successive infections

Ten Holstein female calves, about 6–7 months old, were used. All the animals were tested seronegative and non viraemic for BTV and bovine viral diarrhoea virus (BVDV), and seronegative for bovine herpesvirus 1 (BoHV1). A thorough general clinical examination was carried out on all the animals by a veterinarian before including them in the study, to confirm their asymptomatic state, in accordance to physiological standards [18].

The calves were housed in an insect-secured BSL3 zone at the Experimental Infectiology Platform (PFIE) of the INRA centre of Tours (Nouzilly, France). The local ethical committee approved the experimental protocol (dossier n.2011-10-1). Three groups were created: a group of four non-vaccinated calves (group NV, calves 1–4), a group with four vaccinated calves (group V, calves 5–8), and an environmental control group with two calves (group C, calves 15 and 16). Vaccination against BTV8 was performed using the inactivated commercial vaccine BTVPUR AlSAP 8 (Merial, Lyon, France) following manufacturer’s instructions.

**Figure 1:**
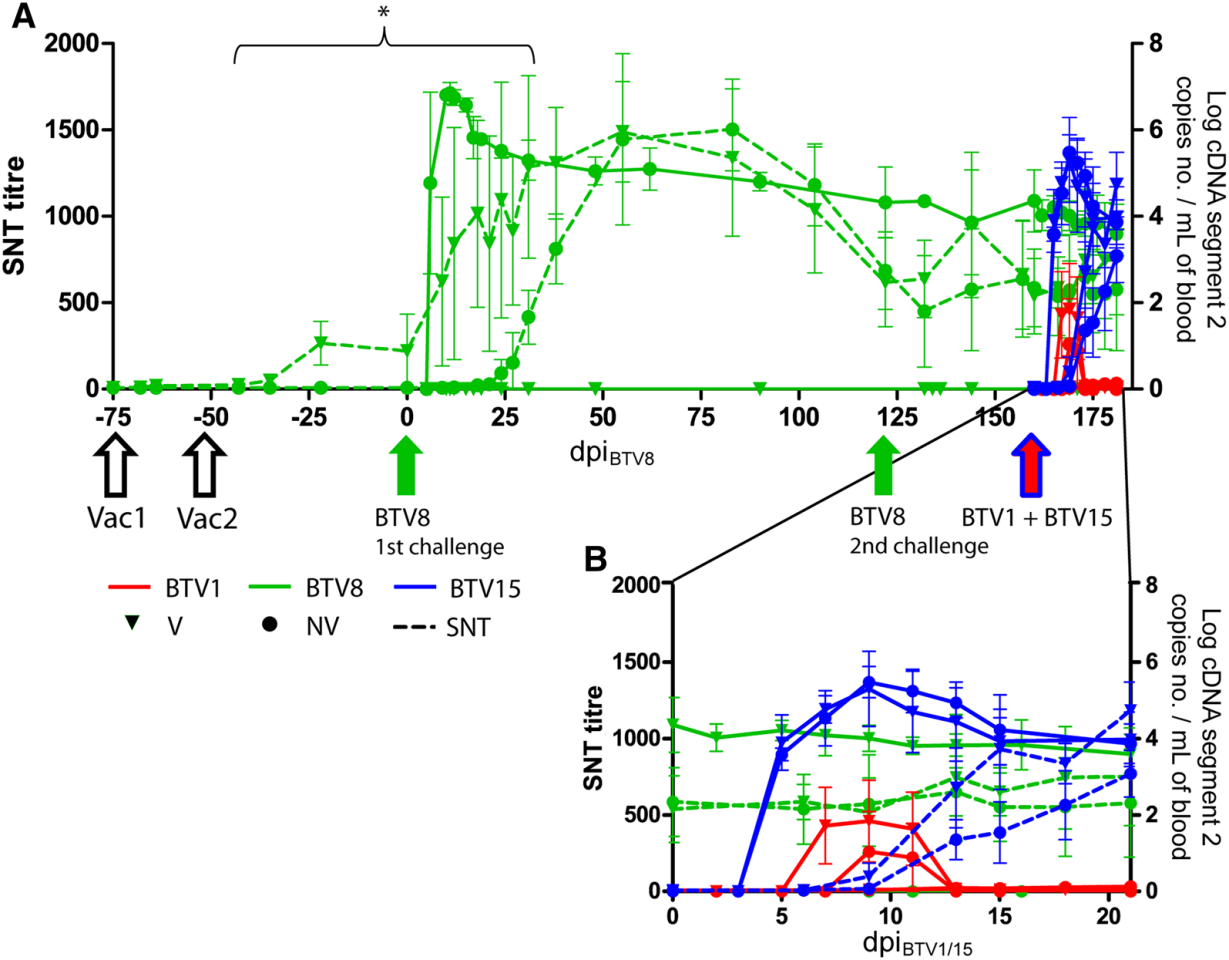
**Neutralising antibodies and viral RNA detection following BTV8 challenges and BTV1/BTV15 superinfection. A** Mean neutralising antibodies titres and mean copy number of VP2 cDNA are shown for vaccinated (V) and non-vaccinated (NV) calves, after vaccinations and challenges with BTV8 and BTV1/BTV15 superinfection. Standard deviation is shown as error bars. The two vaccine injections are represented as arrows followed by Vacc1 and Vacc2 respectively. Infectious challenges are represented as arrow labelled with the corresponding serotypes. * above braces: time period with neutralising Abs titre of V group significantly higher than NV group; *P* < 0.05. **B** Focus on the BTV1/BTV15 superinfection experiment.

Calves of group V and NV were infected twice, 4 months apart, using the same BTV8 inoculum described in a previous experimental infection [19]. Briefly, a calf inoculated with a BTV-8 strain passaged twice in baby hamster kidney fibroblasts (BHK-21) cells (BEL2006/01 BHK-21 P2), was blood sampled at the viraemic peak, showing clinical signs. The first infection took place 50 days after the second vaccine shot, and the second challenge 120 days later (Figure 1A). Each time, half of the dose was inoculated via the jugular vein and half subcutaneously. For each one of these challenges, all eight infected animals were administered a total of 15 mL of blood, corresponding to a titre of 10^3^ embryo lethal dose 50 (ELD_50_).

The animals were daily examined and sampled for EDTA-blood and whole blood on dry tubes during the 3 first weeks after infection, then twice a week until superinfection. For clarification purpose, days post infection regarding BTV8 successive infection are mentioned as dpi_BTV8_, with day of first challenge as dpi_BTV8_ 0.

### BTV superinfection

About 5 months (160 days) after the first BTV8 infection, and 40 days after the second one, calves of groups NV and V were challenged with BTV1 infectious blood, kindly provided by the Friedrich-Loeffler Institute. Each animal received 10^6.15^ tissue culture infective dose 50% (TCID_50_) of virus, half intravenously and half subcutaneously. An incidental contamination of the inoculum with BTV15 was discovered during the course of the study [17].

The animals were daily examined and sampled for EDTA-blood and whole blood on dry tubes until the end of the experiment.

Calves were euthanized 21 days after the superinfection and necropsied (Figures 1A and B). Days post infection for superinfection is mentioned as dpi_BTV1/15_.

### BTV1 and BTV15 single infections

Eight Holstein male calves, about 6–7 months old, were housed in the BSL3 facility, at CODA-CERVA’s (Veterinary and Agrochemical Research Centre, Uccle, Belgium) experimental centre (Machelen, Belgium). These animals fulfilled the same inclusion criteria of the BTV8 successive infections and were naïve for BTV, BVDV and BoHV-1. The experimental protocol was reviewed by the competent authority (Ethical Committee of the Institute of Public Health-Veterinary and Agronomical Research Centre) and subsequently approved (ref. 110228-01 RT 10/10 BLUETONGUE).

After an acclimatization period of 2 weeks, calves were divided in three groups [BTV1: calves 9–11, and BTV15: calves 12–14, single infections; mock-infection: four calves (calves 17–20)]. The infection was performed with a volume of 1 mL of virus diluted in Dulbecco’s modified eagle medium (DMEM, Lonza BioResearch, Belgium), half intravenously and half subcutaneously.

The BTV1 strain has been provided by CODA-CERVA, from sub-saharian origin, derived from the European Community Reference Laboratory for bluetongue at the Pirbright Laboratory, UK collections, and subsequently passaged two times in BHK-21 cells. The infection was performed with 10^6^ TCID_50_ per animal.

BTV15 was provided by CODA-CERVA, derived from the European Community Reference Laboratory for bluetongue at the Pirbright Laboratory, UK and was then passaged twice on BHK21 cells at CODA-CERVA. Calves were infected with 10^4^ TCID_50_.

Mock-infected calves were inoculated with sterile DMEM following the same routes and volume.

The animals were daily examined and sampled for EDTA-blood and whole blood on dry tubes until the end of the experiment. Calves were euthanized 35 dpi and necropsied. Days post infection for single infections are mentioned as dpi_single_.

### Clinical and post-mortem examination

After each challenge, the individual body temperature and the clinical signs were monitored for 3 weeks. The severity of the infection was quantified by calculating clinical scores on a daily basis, leading to overall cumulative clinical scores by groups and animal. For this purpose, a standardised clinical form adapted from Saegerman et al. was used [20]. As BTV1 and BTV15 single infections only involved three animals whereas other groups had four calves each, total clinical score was pondered to allow direct comparison of clinical scores from different experiments.

Samples of spleen, thymus, prescapular and mesenteric lymph nodes were collected from infected and control calves and stored at −80 °C for virus detection.

### Serology

Neutralizing antibodies (Abs) were titrated by seroneutralization (SNT). Two-fold serial dilutions of the sera (1:10–1:1280) were tested in the presence of 100 TCID_50_ of virus, as previously described [21]. The neutralizing antibody titre was defined as the reciprocal of the serum dilution causing a 50% reduction in cytopathic effect. Serum samples with a titre <20, =20 and >20 were considered negative, doubtful and positive, respectively.

In order to identify in vitro cross neutralization between BTV1, BTV8 and BTV15, the serum of the calf infected with BTV8 and showing the highest anti-BTV8 antibody titre was tested in the presence of BTV1 or BTV15. Similarly, the serum of the calves infected with BTV1 and BTV15 following single serotype infections and showing the highest neutralizing antibody titres against the correspondent virus were tested against heterologous serotypes. In vitro cross neutralization was measured using the percentage of neutralization obtained using heterologous serotypes with immune serum and compared to homologous neutralization as reference (100%).

In the course of the two BTV8 challenges, and after BTV1 and BTV15 single infections, seroconversion against VP7 antibodies was also evaluated using a commercial competitive ELISA kit (ID Screen^®^ Bluetongue Competition ELISA kit, ID Vet, France). Results were expressed as % of negativity (PN) compared to the negative kit control and transferred to a positive, doubtful or negative result according to the cut-off settings provided by the manufacturer (PN ≤ 35 is positive; 35 < PN ≤ 45 is doubtful; PN > 45 is negative).

### BTV RNA detection

Viral RNA extraction from the blood was achieved using the QIAamp Viral RNA Mini Kit (Qiagen, Germany). Viral RNA denaturation and reverse transcription followed by qPCR were performed as previously described [22]. BTV RNA was detected by serotype specific RTqPCR, using a fragment of BTV segment 2 as the target. Serial dilutions of in vitro constructed plasmids (pGEM^®^-T Easy Vector, Promega, The Netherlands) carrying the target part of the segment 2, specific for each serotype, allowed the absolute quantification of the viral cDNA equivalent in samples. Quantification was expressed in cDNA copy number/mL of blood. RTqPCR cycling conditions, primers and probes were similar to the ones described by Vandenbussche et al. [22] for BTV1 and BTV8 (RTqPCR_BTV1_S2 and RTqPCR_BTV8_S2, respectively), and Eschbaumer et al. [18] for BTV15 (RTqPCR_BTV15_S2). In all the RTqPCR of this study, bovine beta-actin was contemporaneously amplified as internal control (RTqPCR_ACT) [23].

BTV RNA detection was performed on all the collected organs starting from approximately 100 mg of tissue, which was processed using TRI reagent according to the manufacturer’s instructions (Life Technologies Europe BV, Gent, Belgium). BTV and bovine beta-actin detection were performed by RTqPCR as described above.

### Viral growth assay

In vitro replication of BTV1, BTV8 and BTV15 were compared on VERO and Bovine Pulmonary Endothelial cells (BPAEC). BTV8 was the same as in the BTV8 successive infections, and BTV1 and BTV15 were the same as in single infections. These viruses were used for a growth assay following a protocol previously described [24]. Briefly, VERO and BPAEC confluent cells in 24 wells plates were inoculated with a multiplicity of infection (M.O.I.) of 0.05, and after 0, 8, 24, 48 and 120 h post infection (hpi), supernatant was removed and stored at −80 °C. Each virus underwent three independent assays on each cell type. Supernatants were then titrated by end-point dilution and titres expressed as Log10 of TCID_50_/mL.

### Statistical analysis

Mean cumulative clinical scores were analysed using linear mixed model, with calf as random effect. Viraemia and serological results were compared using two-way ANOVA with repeated measures. RNA detection in organs at necropsy, frequencies and proportions were compared with Fisher’s Exact Test for count data [25]. For all tests, *P* values <0.05 were considered significant. In case of multiple comparisons, a Bonferroni correction was applied to reduce the risk of type I error (conservative approach) and a Holm correction was applied when more than four comparisons had to be tested. Statistical analyses were performed using the R software/environment (R-3.1.2, R Foundation for Statistical Computing, [26]) and SAS software, Version 9.3 TS level 1M2 of the SAS System for Unix, and SAS University Edition (SAS Institute, Cary, NC, USA).

## Results

### BTV8 successive infections

#### Clinical examination

From the beginning to the end of the experiment, control calves and vaccinated animals (V) did not show any clinical signs that could be related to BTV infection. After the first challenge, clinical signs showed by NV calves were slight, mostly consisting in ocular lesions and to a lesser extent by oral lesions. Clinical signs consistent with BTV8 infection could be reported from 7 to 21 dpi_BTV8_. After the second challenge no clinical manifestations or temperature rise could be detected in any animal.

#### Serology

VP7 and anti-BTV8 neutralizing Abs in control calves could not be detected at any tested time points.

First vaccination of the calves did not induce detectable neutralizing Abs, which could only be detected 7 days after the booster vaccination. In NV group, neutralizing Abs were first detected at 18 dpi_BTV8_ (Figure 1A). The titre of anti-BTV8 neutralizing Abs of the V group was significantly higher between −43 dpi_BTV8_ (thus 35 days after the first vaccine shot) and 27 dpi_BTV8_ (*P* < 0.005), and then Abs titres of both NV and V groups followed a similar trend until the end of the experiment (Figure 1A).

Following the second BTV8 challenge, neutralizing Abs titres underwent a boost in both NV and V groups until 33 dpi_BTV8_ (Figure 1A).

**Figure 2:**
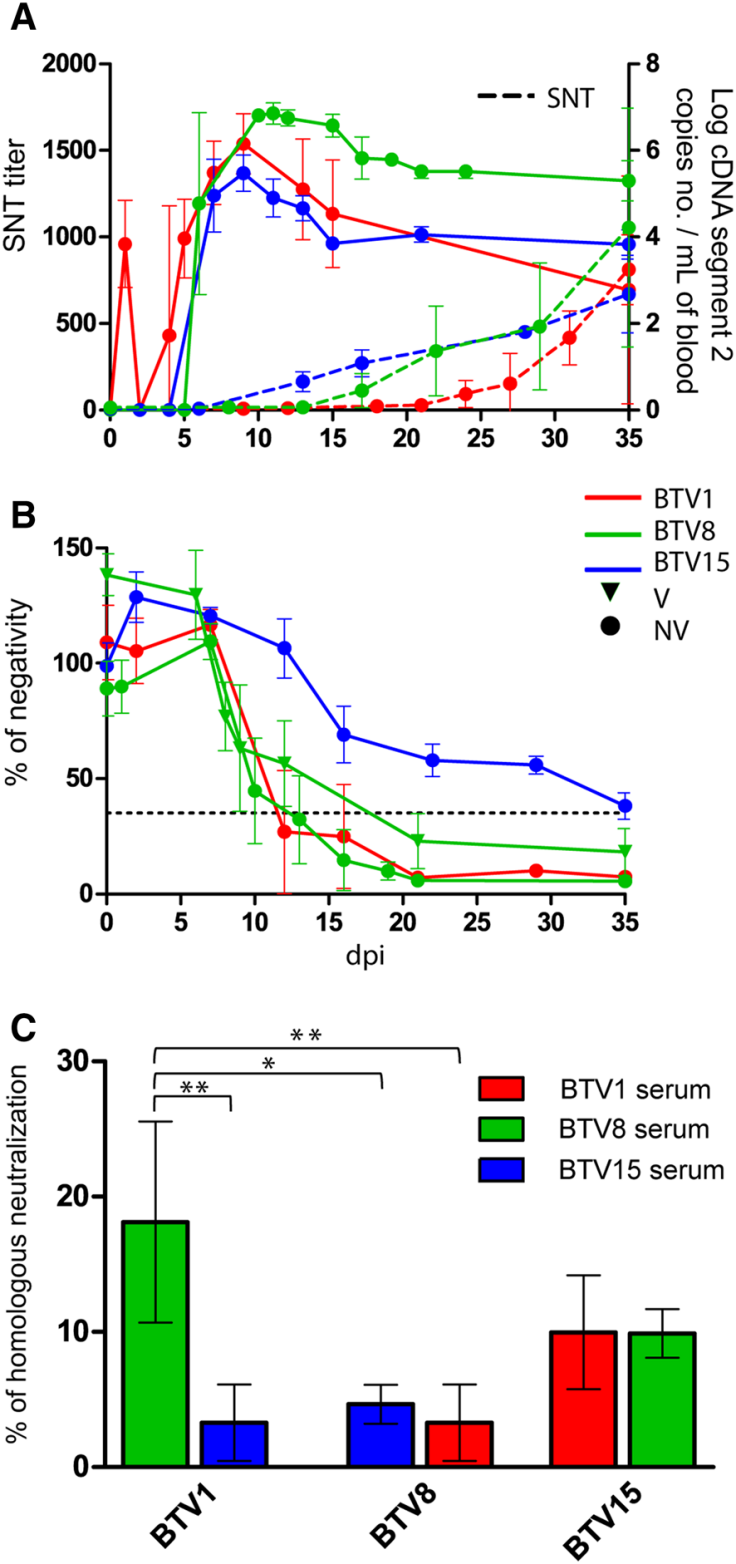
**Serology, RNA detection and cross neutralization results following single infections with BTV1, BTV15 and BTV8. A** Results of RTqPCR and SNT in V and NV groups of calves, after BTV1 and BTV15 single infection challenges compared with BTV8 first challenge in NV group. Dashed lines represent SNT results. **B** cELISA % of negativity following BTV1 and BTV15 single infection, BTV8 vaccination in V group and first BTV8 challenge in NV group. Dotted line represents positivity threshold, with % of negativity <35 considered as positive. **C** Cross neutralization results, with BTV1, BTV8 and BTV15 compared to the respective heterologous immune serum. Results are expressed as a percentage of the highest homologous neutralization titre. *P < 0.05; **P < 0.01.

The use of an ELISA allowed the detection of anti-VP7 Abs in all the vaccinated animals as soon as 3 days after second vaccine injection (Figure 2). Then PN of vaccinated animals did not evolve significantly until the end of the measures at 180 dpi_BTV8_ (Additional file 1). Non-vaccinated calves were confirmed seropositive between 10 and 19 dpi_BTV8_, with no significant variations until the end of the measures at 180 dpi_BTV8_ (Additional file 1, *P* > 0.05).

#### BTV RNA detection

BTV8 RNA was never detected in the EDTA-blood samples of control and vaccinated calves during the course of the two BTV8 infections. In the NV group of calves, BTV RNA could be detected starting from 5 dpi_BTV8_. After the viraemic peak (11–15 dpi_BTV8_) a progressive decrease in BTV8 RNA was measured (Figures 1A and B), until the end of the experiment.

### BTV superinfection

#### Clinical and post-mortem examination

In the NV as well as in the V group, lesions following BTV1/BTV15 superinfection were mainly conjunctivitis with serous to purulent discharge. Erosions of the muzzle, erosions and ulcerations of the gums and the dental pad and later crusts on the muffle or on the cutaneous-mucous junction were commonly notified. Reddening and swelling of the coronal margin and interdigital space were also mentioned. Conjunctivitis and congestion of the lower limb were mild to severe and respectively less severe and absent in the previous BTV8 infection. At the end of the experiment cumulative clinical score of the NV BTV1/BTV15 superinfected group was higher than in NV BTV8 and V BTV1/BTV15 groups, however the difference was not significant (*P* > 0.4, Additional file 2).

**Figure 3:**
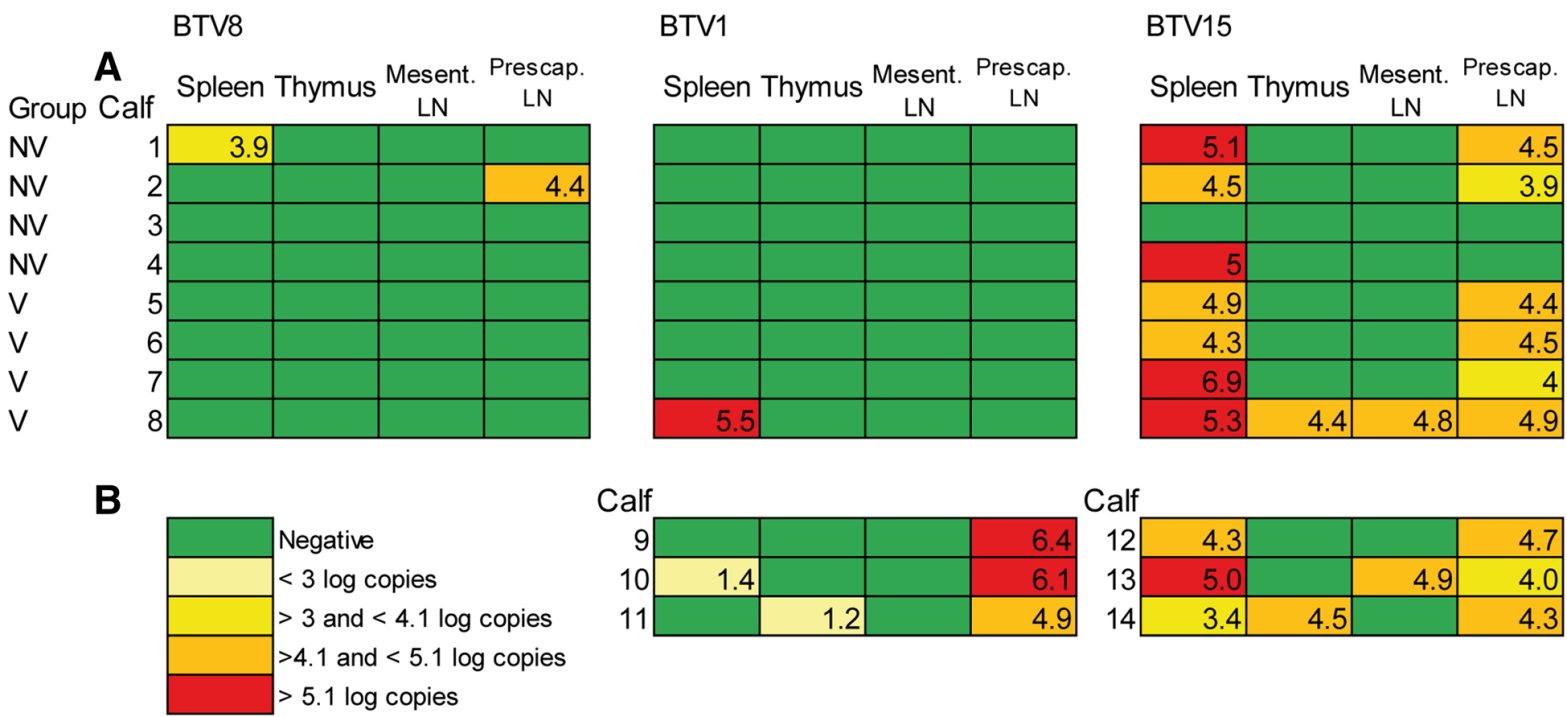
**BTV RNA detection in organs. A** BTV1, BTV8 and BTV15 detection in organs following BTV1/BTV15 superinfection. **B** BTV1 and BTV15 detection in organs following single infections. Results are expressed as Log_10_ copy number/100 mg of tissue. Mesent. LN: Mesenteric lymph node. Prescap. LN: Prescapular lymph node.

The necropsy revealed petechial haemorrhages of limited extent in prescapular and submandibular lymph nodes, and thymus, at least in one of these organs in all the superinfected calves. BTV8 RNA could be detected in prescapular lymph node of one calf and in the spleen of another one, both from NV group; BTV1 RNA could only be detected in the spleen of one vaccinated calf and BTV15 RNA could be detected with a significant higher frequency (*P* < 10^−5^) in 15 organs belonging to 7 different calves (5/15 in NV group and 10/15 in V group; Figure 3).

No BTV RNA could be detected in any of the tested organs from control animals.

#### Serology

After the superinfection, high levels of residual neutralizing antibodies against BTV8 were found throughout the experiment in all the infected animals, with a roughly steady level (Figures 1A and B). There were no significant differences between NV and V groups through time in anti BTV8 neutralizing antibodies (two-way ANOVA with repeated measures, group effect: *P* = 0.78, group time interaction: *P* = 0.84). By contrast, BTV1 only gave rise to very low titres of neutralizing antibodies.

BTV15 neutralizing antibody titre increased regularly after infection, with a positive titre detectable in most of the animals at 9 dpi_BTV1/15_ (Figure 1B). BTV15 neutralizing Abs followed an increasing trend until the time of euthanasia; in previously vaccinated animals a higher and significant earlier raise of neutralizing antibody was found compared to non-vaccinated ones (two-way ANOVA with repeated measures, group effect: *P* < 0.02, group time interaction: *P* < 2 × 10^−4^) (Figure 1B).

#### BTV RNA detection

At the time of the superinfection, a residual BTV8 RNAemia was detected in the NV group of calves. BTV8 RNAemia decreased through the time of the experiment, but was still detectable in all the NV animals by the time of euthanasia (180 days after the first BTV8 challenge—Figure 1).

The superinfection inoculum contained respectively 10^6.8^ and 10^7.6^ copies of segment 2 cDNA per mL of blood, for BTV1 and BTV15.

After superinfection in the NV and the V groups, BTV1 could only be detected inconstantly, from one to 3 days amongst all the tested day-points and at lower copy number than BTV15 (Figure 1B). On the contrary, BTV15 could be easily detected in both groups and among all the infected calves. RNAemia through time was significantly different between BTV15 and BTV1 whichever the considered vaccination status of the animals (*P* < 10^−8^). Mean copy number at viraemic peak was 10^5.4^ (±0.7 Log) cDNA copy number/mL of blood for BTV15 and 10^2.4^ (±1.8 Log) for BTV1. BTV15 could be detected until the end of the experiment (Figure 1B). Between V and NV groups, detection of BTV1 and BTV15 was not significantly different (*P* = 0.18 and *P* = 0.86 for BTV1 and BTV15 respectively).

No viral RNA could be detected in control animals (data not shown).

### BTV1 and BTV15 single infections

#### Clinical and post-mortem examination

During BTV1 single infection, one calf underwent sporadic hyperthermia and all the three calves of the group had mild oral and ocular lesions. No systemic impact was reported in any of these animals.

During BTV15 single infection, infected animals showed very mild clinical conditions compatible with BT, including congestion and crusts on the nostrils and oral mucosa. One calf showed hyperthermia at 7 dpi (39.6 °C) and at 14 (40 °C) and 15 dpi (40.7 °C), with no other lesion throughout the experiment (Additional file 2). No hyperthermia was recorded in any of the other cattle at any stage of the experiment.

Overall, the sum of clinical scores in BTV1 and BTV15 single infected groups were not significantly different when compared to control animals or to the V group after BTV8 challenge (*P* > 0.14), but were significantly lower when compared to first BTV8 challenge in NV calves and the superinfection with BTV1/15 inoculum in both V and NV groups (*P* < 0.002).

Necropsy for BTV1 single infection revealed moderate petechial haemorrhages in mesenteric and mediastinic lymph nodes in one calf, prescapular and mediastinic lymph nodes in another one and no lesions in the last one.

In the calves with BTV15 single infection, no BTV specific lesions could be found at necropsy. Petechial haemorrhages were reported on the thymus and the prescapular lymph node of two calves, respectively, in both cases on a limited amount.

All the three BTV1 infected calves had a positive BTV1 detection in prescapular lymph nodes. In addition, viral RNA could be detected in the thymus and spleen of two other calves, respectively (Figure 3).

RTqPCR revealed BTV15 positive detection in the spleen and the prescapular lymph nodes of all of the three BTV15 infected calves. In addition, thymus and mesenteric lymph node were shown to be positive in two calves, respectively (Figure 3). The frequency of positive detection in organs was not different between BTV1 and BTV15 (Fisher’s Exact Test for count data, *P* > 0.4).

No specific lesions or BTV RNA detection could be found in control animals. A few non-specific abscesses could be found in the lung of one of these calves.

#### Serology

During BTV1 single infection anti-BTV1 neutralizing antibodies could be detected for the first time at the 16 dpi_single_ tested time point and then increased regularly until the end of the experiment (Figure 2A). Similarly, also anti-BTV15 neutralizing antibodies were measured starting from 16 dpi_single_ in the course of BTV15 single infection and the titres increased regularly until the time of euthanasia (Figure 2A).

There was no significant difference between SNT titres of BTV1 and BTV15 single infection and BTV8 after first infection in NV group during the first 35 days (*P* > 0.24).

BTV1 infected calves seroconverted regarding anti-VP7 antibodies between 7 and 16 dpi_single_, and were still all seropositive at the end of the experiment.

Anti-VP7 Abs in BTV15 infected animals clearly increased between 10 and 15 dpi_single_ in all infected calves (Figure 2B). However, only 1/3 calves seroconverted at the end of the experiment at 35 dpi_single_, whereas the two other calves remained slightly out the positivity limit (mean PN = 38 ± 5.7, Figure 2B). At 35 dpi_single_ PN of BTV15 infected group was significantly higher when compared to BTV1 single infection group and BTV8 groups at 35 dpi_BTV8_ (*P* < 0.007).

In vitro cross neutralization assay only showed limited cross reactivity between BTV8 immune serum against BTV1 virus (18% ± 7.4% of the BTV8 immune serum homologous neutralization, Figure 2C). However, this cross reactivity was significantly higher than the one measured between BTV1 versus BTV15 immune serum, BTV8 versus BTV1 and BTV15 immune serums (*P* < 0.02). BTV1 and BTV8 immune serum elicited a limited cross reactivity toward BTV15 (10% ± 4.5% and 10% ± 1.8%, respectively).

#### BTV RNA detection

In the course of BTV1 single infection BTV RNA could be detected as soon as 1 dpi in all the calves of the group, but then could only be detected again at 2 and 3 dpi for respectively one and two calves.

During BTV15 single infection viral RNA could be detected starting from 7 dpi in all three calves. BTV15 RNA could be detected until the end of the experiment (Figure 2A). The levels of RNA peaked in the blood of both BTV1 and BTV15 groups between 9 and 11 dpi_single_. BTV1 cDNA copy number detected in single infected calves was significantly higher than in BTV1 superinfected ones, no matter their vaccination status (*P* < 10^−4^). By contrast, there were no significant differences in cDNA copy numbers between BTV15 superinfected (V and NV groups), BTV1 and BTV15 single infected calves (*P* > 0.2). Moreover, BTV1 and BTV15 cDNA copy numbers were not significantly different from BTV8 cDNA copy number during first infection in NV group (*P* > 0.13).

### Viral growth assay

**Figure 4:**
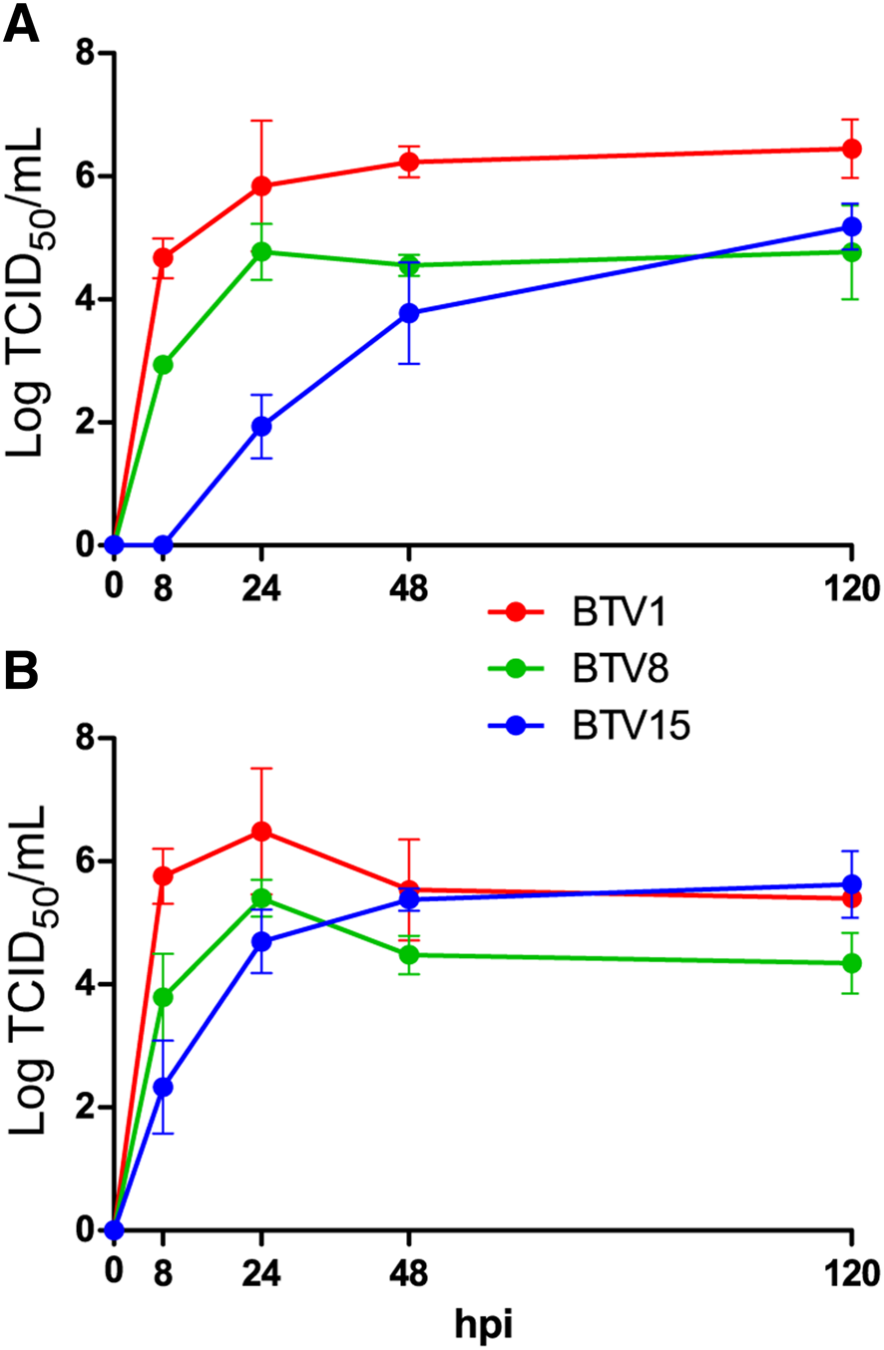
**In vitro growth kinetics of BTV1, 8 and 15 on VERO and BPAEC cells.** VERO cells (**A**) and BPAEC (**B**) were infected at a multiplicity of infection (M.O.I.) of 0.05 and supernatants collected at 8, 24, 48, and 120 h post infection (hpi). Viruses were the same as used for single infection (BTV1 and BTV15) and BTV8 was the same as in the BTV8 successive infection experiment. Supernatants were then titrated on VERO cells by end-point dilution and the virus titres expressed as log_10_ TCID_50_/mL. Three different assays were performed independently, each time involving the three serotypes, tested on the different cell lines. Standard deviations are displayed as error bars.

From 0 to 24–48 hpi, BTV1 showed a faster replication, however not significant, whichever the considered cell line (Figure 4). In VERO cells BTV15 grew less efficiently than BTV1 from 0 to 48 hpi (*P* < 10^−5^), but finally reached by 120 hpi similar titres to BTV1 and BTV8 in VERO cells (*P* > 0.14) and to BTV1 in BPAEC (*P* > 0.9). Homologous viral growth was not significantly different between cell types (*P* = 0.15), and there was no significant differences between serotypes in BPAEC (*P* > 0.3).

## Discussion

The influence of the existent active immunity towards the European BTV8 strain on the outcomes of a superinfection with BTV1 was evaluated. The BTV1 inoculum appeared to be contaminated with BTV15 thus the animals were actually infected with a mixed BTV1–BTV15 inoculum. BTV8 active immunity was evaluated either by vaccination followed by infectious challenges or by infectious challenges alone. Two successive infections with the same BTV8 strain were realized 4 months apart. In line with field data [9], vaccination only elicited the production of neutralizing Abs detectable after the second vaccine boost. Vaccinated animals underwent a significantly earlier detection of neutralizing antibodies after the first BTV8 challenge when compared to non-vaccinated calves. In the NV BTV8 group, the detection of BTV8 RNA lasted until 180 dpi_BTV8_ (end of the experiment), which is consistent with currently existing literature data [27, 28]. Non-vaccinated calves infected with BTV8 showed a slight to mild clinical picture. A moderate impact of the disease caused by BTV8 on cattle is not unusual, in experimental infections [22, 29] as well as in the field [30–32].

After the unexpected contamination of the BTV1 inoculum with BTV15, the influence of both viruses on the outcome of the infection was investigated. In the inoculum used for the superinfection, the copy number of BTV1 segment 2 cDNA per mL of inoculum was about tenfold lower than BTV15. After superinfection BTV1 could be found irregularly and only at a few tested time-points in the blood of the calves while BTV15 was detected with high levels of RNAemia until the end of the experiment in both V and NV groups. The overwhelming replication of BTV15 versus BTV1 is in line with results reported by Eschbaumer et al. [18]. Domination of one serotype on another during mixed infection has been previously reported [33] and the same authors observed that about 5% of progeny viruses were actually reassortants. Any genome segment can be involved in reassortment which is readily generated, as demonstrated by Shaw et al. [34]. As in the current study viral RNA was based on segment 2 quantification, it is not possible to rule out that some of the segment 2 detected by RTqPCR being actually part of reassortant viruses. This was also one of the hypotheses brought to light by Dal Pozzo et al. [25] to explain the predominance of BTV8 on BTV1 and BTV15 in the course of a triple co-infection. Another possible explanation is the viral interference, occurring between two or more viruses infecting simultaneously the same host.

BTV8 was recently used to study underlying IFN-I control mechanisms by the virus [35]. To investigate whether the different tested serotypes would also show different replication patterns in vitro or not, growth curves of BTV1, BTV8 and BTV15 were established in two common cell lines, BPAEC and VERO cells. VERO cells are deficient for IFN-I production [36]. BTV15 did not replicate as much as BTV1 in VERO cells during the first 48 h. Nevertheless, in IFN-I competent cells such as BPAEC primary line, both BTV serotypes replicated following a similar pattern through time. These results are not inconsistent with the hypothesis of BTV15 to be better adapted to IFN-induced state when compared to BTV1, possibly explaining the relatively more efficient replication of BTV15 in vivo or in vitro in IFN-competent cell lines. Another hypothesis to explain the difference between BTV1 and BTV15 RNAemia after superinfection might be related to the influence of BTV8 immunity on these two serotypes. The level of heterologous reactivity as assessed by SNT between BTV8 immune serum and BTV serotypes 1 and 15 was low, yet higher for BTV1 (18 ± 7.4% versus 10 ± 1.8% for BTV1 and BTV15, respectively; Figure 2C). This result is in line with Hund et al., which reported partial cross neutralization between BTV8 positive serum and BTV1, despite the genetic distance between these serotypes [9].

In addition, no significant difference was reported between RNAemia of BTV15 superinfected calves (from V and NV groups, both immunized against BTV8) and RNAemia of BTV15 single infected calves. Thus this low in vitro humoral cross reactivity between BTV8 immune serum and BTV15 seemed to have no significant influence on BTV15 RNAemia in vivo in contrast to the BTV1 RNAemia.

BTV1/BTV15 superinfection led to clinical disease in both V and NV animals. On the contrary, during BTV1 and BTV15 single infection the calves had very low clinical scores. The reason of this difference remains uncertain; however individual variability could be part of the explanation.

After superinfection, BTV1 neutralizing Abs only reached very low levels, as a consequence of the very low BTV1 RNA detection. By contrast, BTV15 neutralizing Abs extended to high titres, either in BTV1/BTV15 superinfected animals or in BTV15 single serotype infected calves.

Despite high neutralizing Abs detection following BTV15 single serotype infection, ELISA detecting VP7 Abs showed a mean PN at 35 dpi just above the positivity threshold. This is consistent with previous reports showing that significant immunological differences exist between BTV15 and other BTV serotypes and that monoclonal antibodies raised against BTV1 VP7 failed to react with BTV15 VP7 [37]. When assessing diagnostic tools aiming at non-serotype specific detection, it would be therefore advisable to include distantly related strains in the test panel to cover most of the genetic variability displayed by BTV proteins.

Vandenbussche et al. suggested to use the ID Vet cELISA kit with a cut off of 66 PN instead of 35 for BTV8, as recommended by the manufacturer, to achieve optimal accuracy for both screening and diagnostic [38]. Taking into account this suggestion, all of the three calves would have been considered as seropositive by day 21 post infection with BTV15.

Unlike the European BTV8, known for its increased virulence in bovine, BTV1 and BTV15 have been associated with subclinical or very mild disease in this species. Numerous factors are known to influence the severity of BT in individual ruminants; nutritional status, immune status and age, breed, environmental stresses such as high temperature and ultraviolet radiation [39]. In this study, the accidental co-infection with BTV1 and BTV15 and the obtained severe clinical outcome underlined the potential higher pathogenicity of a co-infection.

The main objective of this study was to observe the outcomes of a superinfection in calves previously immunized with the European BTV8. BTV1 or BTV15 RNA detection in superinfected animals was not different whether BTV8 immunization was acquired through vaccination and challenges or challenges alone. Furthermore, a low cross neutralization was measured between BTV8 and BTV1, and between BTV8 and BTV15. Taken all together in the context of the European BT epidemiological situation, the results could suggest that an infection or a vaccination with the European BTV8 strain would not efficiently protect the bovines from a superinfection with the BTV1 or BTV15 strains used in the study.

## Additional files

10.1186/s13567-016-0357-6 **BTV group specific anti-VP7 antibodies, after vaccination against BTV8 and BTV8 challenges, for non-vaccinated, control and vaccinated calves.** BTV group specific anti-VP7 antibodies as the % of negativity. Dashed line represents the cut off value. A % of negativity under the cut off (35%) is considered positive. NV: non vaccinated; V: vaccinated. The two vaccine injections are represented as arrows labelled Vac1 and Vac2 respectively, and first and second BTV8 challenges are represented as green arrows. Standard deviations are represented as error bars.
10.1186/s13567-016-0357-6 **Cumulative clinical scores.** Cumulative clinical scores after the first BTV8 challenge in NV and V animals, BTV1/BTV15 superinfection and BTV1 and BTV15 single infections. Sd: Standard deviation.
