# Supplementary figures and images for: Experimental bluetongue virus superinfection in calves previously immunized with bluetongue virus serotype 8
Source: Vet Res. 2016 Jul 28;47:73. doi: 10.1186/s13567-016-0357-6 (PMC4964278; doi:10.1186/s13567-016-0357-6)

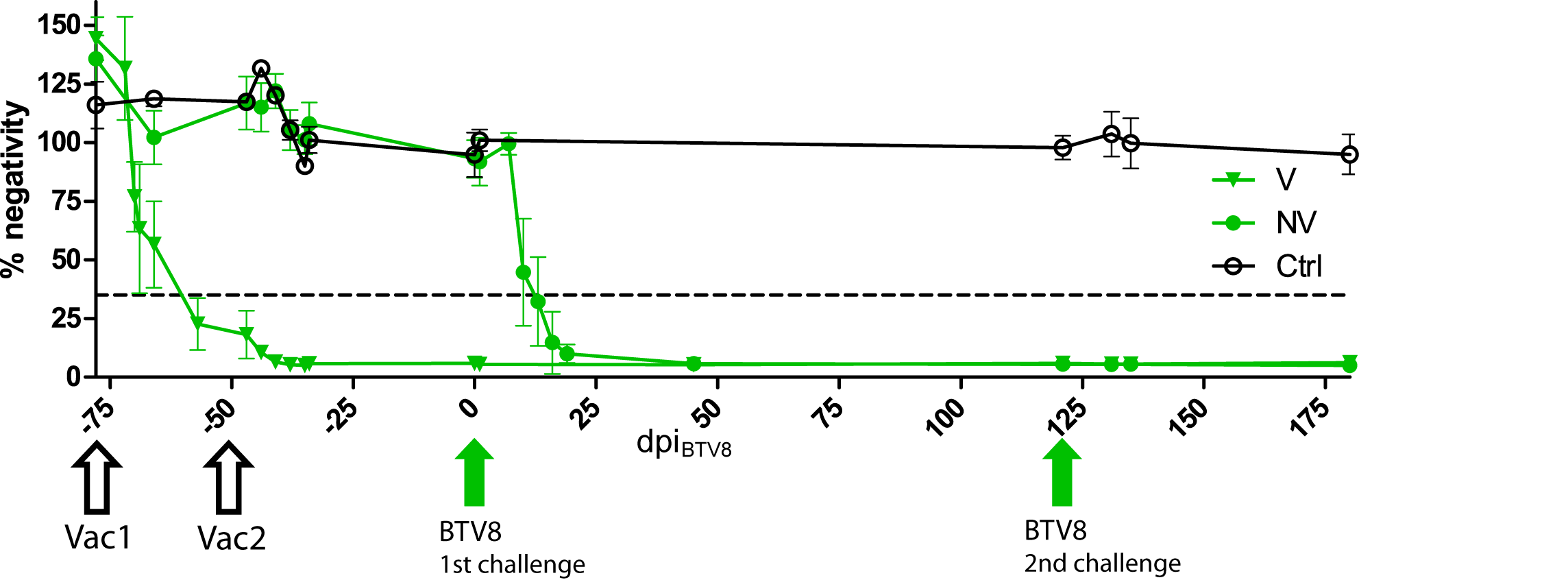

Supplement: Supplementary file 1 — 10.1186/s13567-016-0357-6 BTV group specific anti-VP7 antibodies, after vaccination against BTV8 and BTV8 challenges, for non-vaccinated, control and vaccinated calves. BTV group specific anti-VP7 antibodies as the % of negativity. Dashed line represents the cut off value. A % of negativity under the cut off (35%) is considered positive. NV: non vaccinated; V: vaccinated. The two vaccine injections are represented as arrows labelled Vac1 and Vac2 respectively, and first and second BTV8 challenges are represented as green arrows. Standard deviations are represented as error bars. [file 13567_2016_357_MOESM1_ESM.tif]
